# Dynamic X-ray speckle-tracking imaging with high-accuracy phase retrieval based on deep learning

**DOI:** 10.1107/S2052252523010114

**Published:** 2024-01-01

**Authors:** Fucheng Yu, Kang Du, Xiaolu Ju, Feixiang Wang, Ke Li, Can Chen, Guohao Du, Biao Deng, Honglan Xie, Tiqiao Xiao

**Affiliations:** a Shanghai Institute of Applied Physics, Chinese Academy of Sciences, Shanghai 201204, People’s Republic of China; b Shanghai Synchrotron Radiation Facility/Zhang Jiang Lab, Shanghai Advanced Research Institute, Chinese Academy of Sciences, Shanghai 201800, People’s Republic of China; c University of Chinese Academy of Sciences, Beijing 100049, People’s Republic of China; d Zhejiang Institute of Metrology, Hangzhou 310063, People’s Republic of China; SPring-8, Japan

**Keywords:** dynamic X-ray imaging, phase retrieval, speckle tracking, deep learning, computed tomography, X-ray microscopy, phase contrast X-ray imaging

## Abstract

Speckle-tracking X-ray imaging is an attractive candidate for dynamic X-ray imaging owing to its flexible setup and simultaneous yields of phase, transmission and scattering images. However, traditional speckle-tracking imaging methods suffer from phase distortion at locations with abrupt changes in density, which is always the case for real samples, limiting the applications of the speckle-tracking X-ray imaging method. In this paper, we report a deep-learning based method which can achieve dynamic X-ray speckle-tracking imaging with high-accuracy phase retrieval. The calibration results of a phantom show that the profile of the retrieved phase is highly consistent with the theoretical one. Experiments of polyurethane foaming demonstrated that the proposed method revealed the evolution of the complicated microstructure of the bubbles accurately. The proposed method is a promising solution for dynamic X-ray imaging with high-accuracy phase retrieval, and has extensive applications in metrology and quantitative analysis of dynamics in material science, physics, chemistry and biomedicine.

## Abstract 2

A deep-learning based speckle-tracking imaging method is developed, and high-accuracy phase retrieval is successfully achieved with a single shot.

## 1. Introduction

With the development of digital X-ray detectors and high-brightness X-ray sources, dynamic X-ray imaging is extensively utilized in many research fields at millisecond, microsecond, nanosecond and picosecond time scales (Yashiro *et al.*, 2017 ▸; Yu *et al.*, 2022 ▸; Xie *et al.*, 2019 ▸). With the venture of X-ray free-electron lasers (XFELs), femtosecond X-ray imaging is also developing quickly (Kang *et al.*, 2017 ▸). Absorption contributes mainly to the contrast mechanism for dynamic X-ray imaging. With the development of third- and fourth-generation synchrotron radiation facilities (SRFs), phase contrast X-ray dynamic imaging has been developed and applied to dynamic systems including chemical reactions, *in vivo* research of biomedical systems, phase transitions of materials *etc.* (Gradl *et al.*, 2018 ▸). Combined with microtomography, 3D dynamic X-ray imaging is also applicable either in absorption contrast or in phase contrast (Parker *et al.*, 2021 ▸; Xu *et al.*, 2016 ▸; Cao *et al.*, 2018 ▸; Liu *et al.*, 2020 ▸; Chengjie *et al.*, 2017 ▸). To track the moving components in a complex system, move contrast X-ray imaging is developed to eliminate the interference of microstructure variation and high-frequency noises associated with *in vivo* research (Wang *et al.*, 2020 ▸). Unlike the large-scale SRF or XFEL facilities, microfocus X-ray tubes can also be employed for phase contrast X-ray imaging owing to their sufficient spatial coherence. Due to low flux density, this tube-based X-ray imaging system can only be applied to cases with relatively low temporal or spatial resolution.

Due the fact that all the detectors are sensitive only to the intensity of the X-rays and absorption is proportional to intensity, dynamic X-ray imaging is complemented mainly in absorption contrast. As for phase contrast imaging, further efforts are required to retrieve the phase information from the intensity distribution. Among all the phase contrast imaging methods, in-line or propagation-based phase contrast X-ray imaging is most frequently employed for dynamic imaging by directly employing the local-interference-introduced edge-enhancement effect to image the outline of the soft tissues or low-*Z* materials. Phase retrieval to the in-line outline images can roughly differentiate the materials in the sample (Paganin *et al.*, 2002 ▸; Chen *et al.*, 2011 ▸). Therefore, diffraction-based methods including the crystal and grating are developed to retrieve the phase accurately (Ando *et al.*, 2014 ▸; Birnbacher *et al.*, 2021 ▸). Recently, speckle modulation was introduced to supplement X-ray diffraction wth precision optical elements of crystals or gratings and retrieve the phase by speckle-tracking or speckle-scanning (Morgan *et al.*, 2012 ▸; Berujon *et al.*, 2012 ▸). As is well known, speckle-scanning can retrieve the phase accurately but multiple scanning prevents dynamic phase contrast X-ray imaging (Berujon & Ziegler, 2015 ▸; Wang *et al.*, 2016 ▸, Zdora *et al.*, 2020 ▸).

Speckle tracking requires only a single exposure to retrieve the phase and certainly shows promise to achieve dynamic X-ray imaging. Though efforts have been made to refine the phase retrieval, the accuracy of the retrieved phase has been difficult to improve significantly, especially for samples with complex structures (Wang *et al.*, 2015 ▸; Zanette *et al.*, 2015 ▸; Paganin *et al.*, 2018 ▸). A double-exposure method is proposed to reconstruct the phase accurately. However, an additional in-line outline image is needed to implement the phase retrieval (Wang *et al.*, 2017 ▸; Yu *et al.*, 2021 ▸). This means that dynamic X-ray imaging with this method is still not practical. In this paper, deep learning is employed to generate the in-line outline image from the speckle image in the double-exposure method. In this way, the double-exposure method can only be implemented by a single exposure of the speckle image and high-accuracy phase retrieval is anticipated. As a result, dynamic X-ray speckle-tracking imaging with accurate phase is feasible. Firstly, we introduce the principles underlying this deep-learning based method. We then verify the method by experiments with a plastic sponge, a phantom and dynamic foaming. Finally, discussions and conclusions are given.

## 2. Methods

### 2.1. Principle

**Figure 1:**
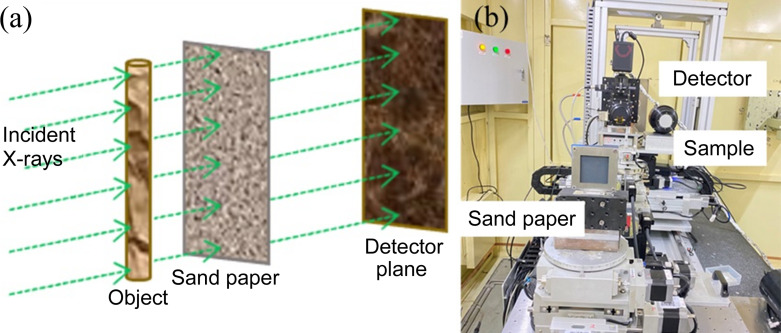
Experimental setup for the single-shot speckle-tracking X-ray imaging: (*a*) schematic diagram, (*b*) photograph of the facilities.

A schematic of X-ray speckle-tracking phase contrast imaging is shown in Fig. 1 ▸. The X-ray beam passes through the sandpaper to generate the speckle pattern. Then, the speckle pattern is modulated by the internal microstructure of the sample. After propagation over a certain distance in free space, the light fields are recorded by an X-ray CCD or CMOS detector. The intensity distribution at the detector plane is expressed as (Wang *et al.*, 2017 ▸)

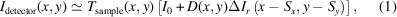

where *T*_sample_(*x*, *y*) is the transmission of the sample, which is actually the intensity distribution of the in-line phase contrast image. *I*_0_(*x*, *y*) is the average intensity of the recorded image field and *D*(*x*, *y*) is the dark-field signal. *I_r_* (*x*, *y*) is the speckle-only pattern and its spatial fluctuation is Δ*I_r_*. *S_x_* and *S_y_* are displacements due to X-ray refraction in the sample. In the double-exposure method, an additional in-line phase contrast image needs to be recorded to restore the phase with high accuracy at locations with an abrupt change of density in the sample, which prevents the method from imaging dynamic processes. In this paper, we introduce deep learning with CGAN to generate the target of an in-line phase contrast image from the recorded speckle pattern of the sample, which needs to be additionally recorded with the double-exposure method. In this way, double-exposure speckle-tracking imaging is achieved with only a single shot. As a result, dynamic X-ray speckle-tracking imaging with high-accuracy phase retrieval can be achieved.

### 2.2. Network structure of deep learning

Throughout the past years, deep learning has seen a widespread adoption in image reconstruction, such as optical imaging (Li *et al.*, 2018 ▸), holographic imaging (Wang, Lyu & Situ, 2018 ▸), super-resolution microscopy (Ouyang *et al.*, 2018 ▸) and phase unwrapping (Wang *et al.*, 2019 ▸). Deep learning is also used to speed up the image reconstruction in a mask-based speckle-tracking method directed at real time X-ray phase contrast imaging (Qiao *et al.*, 2022 ▸). In this paper, deep learning with conditional generative adversarial nets (CGANs) is adopted to generate the target of an in-line phase contrast image from the input of the speckle image recorded at the detector plane. Although convolutional neural network (CNN) is widely used in deep learning, it usually needs the loss function to be set manually. In contrast, CGAN automatically learns the loss function adapted to the data. Thus, it can be applied to a variety of tasks, which need various loss functions, and is well suited to image-to-image translation tasks. Although the training process is time-consuming and data hungry, only one training effort is needed. Once the model parameters are trained, there is no need to retrain and the model can be reused for different cases.

CGAN-based image-generation models have been widely applied in various fields (Li, Li *et al.*, 2020 ▸; Li, Zhang *et al.*, 2020 ▸; Sargent *et al.*, 2020 ▸). Due to advantages in generating large-sized and high-definition images, model pix2pixHD (Wang, Liu *et al.*, 2018 ▸) is adopted for the main architecture of CGAN used in this paper. It consists of two components: generator G and discriminator D. The generator mainly comprises three components: a down-sampling module, a residual module and an up-sampling module. The input layer is a 64 convolution kernel with stride 1 and kernel size 7. The down-sampling module contains four convolutional layers with stride 2 and kernel size 3. The residual module consists of nine residual blocks. The residual block contains two convolutional layers with kernel size 3 and stride 1. As for the up-sampling module, it consists of four transposed convolutional layers with kernel size 3 and stride 2. Note that the convolutional layer of the above-mentioned modules follows Instance Normalization (Ulyanov *et al.*, 2016 ▸) and ReLU layers. The output layer is a convolutional layer with kernel size 7 and stride 1. Then Tanh activation was used to normalize the outputs to −1 or 1. An input image of 1024 × 512 resolution passed the G network and finally output an image of 1024 × 512 pixels. Connecting a local-enhancer can train an image 2048 × 1024 pixels in size, but requires at least 24 GB memory for the GPU.

As for the discriminator, two discriminators, referred to as D1 and D2, which have the same network structure, were used to train *G*(*x*) and *Y* at different scales. The discriminator D1 is trained by the original scale *G*(*x*) and *Y* to obtain detailed information. We down-sample *G*(*x*) and *Y* by a scale factor of 2 to train the discriminator D2 to enlarge the receiving field and generate a globally consistent image. The structure of the discriminator starts with a Conv-leaky ReLU block followed by four conv-Instance norm-leaky ReLU blocks, each of the convolutional layers has kernel size 4 and stride 2. The output layer is a convolutional layer with kernel size 4 and stride 1, which is followed by the Sigmoid neuron. *G*(·) and *D*(·) are defined as mapping functions of the generator and discriminator, respectively. The loss function of conditional CGAN is defined as

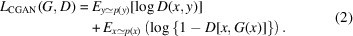

CGAN learns to map a known image *X* to an output image *Y* via a maximized operator, where *G* aims to minimize *L*_CGAN_ while *D* tries to maximize it. Here a double-scale discriminator is adopted, hence the objective refers to

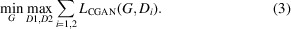

We also used a perceptual loss to match the features in a visual perception network which is used to generate the image required. The perceptual loss could help the model to emphasize contents. VGG19 is leveraged to be a visual perceptual network and denoted ψ. The perceptual loss is expressed as

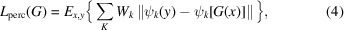

where *E*_*x*,*y*_ is the average, ψ_*k*_ represents the *k*th layers of convolution in the network ψ and *W*_*k*_ is the hyper-parameter used to balance the contribution of the corresponding layer to the loss. Here, *W*_*k*_ from *k* = 1–5 is assigned the values 1/32, 1/16, 1/8, 1/4, 1. Therefore, the final objective is

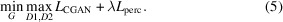

The hyper-parameter controls the impact of perceptual loss and is set to 10 here.

### 2.3. Training strategy

**Figure 2:**
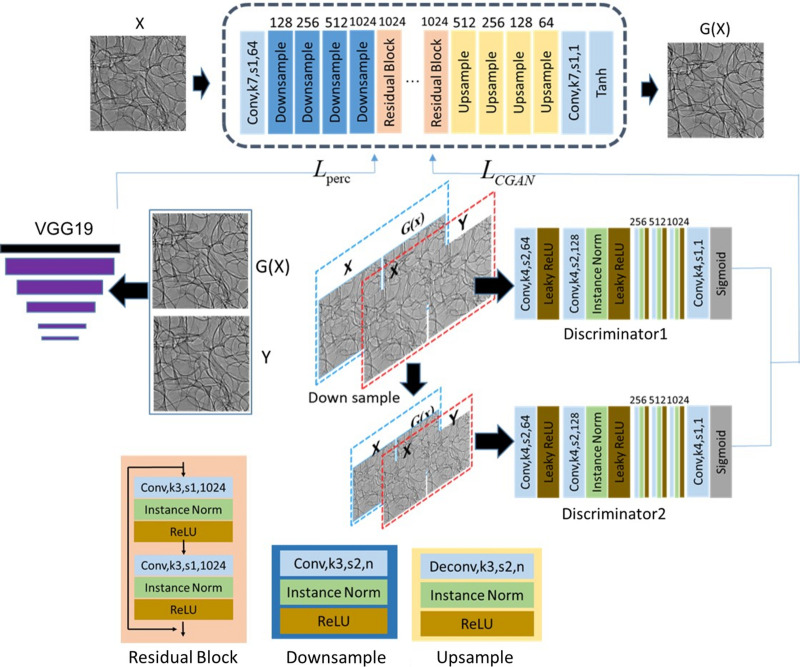
Architecture of the CGAN used for phase retrieval, where Conv,k3,s1,*n* means a convolution with kernel size 3, stride 1, and *n* indicates that the convolution (or deconvolution) has *n* filters.

The training strategy is shown in Fig. 2 ▸. The input of CGAN is an image containing information of the second-order phase and first-order speckle displacement, obtained by subtracting the speckle-only image from the speckle-sample image. The counterpart in-line phase contrast image is used as the target to train the network, where the target is the desirable output of the net for the input image.

For different types of test sample, the network needs to be trained again to obtain new parameters, which means that the network works well with samples with their class of images included in the training dataset. For an artificial sponge, the training dataset is obtained from sponges with similar structures. As for the artificial phantom, phantoms with different sizes and different arrangements were taken as the training dataset. In dynamic polyurethane foaming experiments, we used images collected from fully foamed, stationary polyurethane foam as the training dataset. During CGAN training, the training dataset needs to be arranged as a pair of input and target images, and then the trained network is applied to the imaging of the dynamic foaming processes. During the acquisition of the training dataset, the samples were installed on a rotation table to obtain their projections from different angles, and hence to ensure the diversity of the dataset. In S2 of the supporting information we supply a few pieces of the training dataset as examples to illustrate the training process more intuitively.

We firstly collect several groups of projection images of the sample, which is in the same class of the test sample, with and without sandpaper. Since the input layer that the network accepts is 1024 × 512 pixels, we crop the original image of 2048 × 2048 pixels at random positions before training. In this way, the diversity of the training dataset remains. Note that the crop position of the input and target images needs to be one-to-one correspondence. A total of 2880 pairs of input–target images were used to train the network. The buffer size and batch size were set to 1, the number of epochs was 100. About 30 h were needed to accomplish the training.

## 3. Experimental results

Experiments were carried out at the BL13HB X-ray imaging beamline of the Shanghai Synchrotron Radiation facility (Xie *et al.*, 2020 ▸), with the photon energy of the X-ray beam set to 15 keV. A piece of sandpaper with an average grain size of 8.6 µm was selected as a wavefront modulator to generate the speckle pattern. The test sample was mounted on a motorized rotation stage located 34 m from the super-bending magnet source. The detector was placed 60 cm downstream from the sample, a 2⋄ microscope objective lens was used to collect the visible fluorescence on the scintillator and finally a CMOS camera with a pixel size of 6.5 µm was employed to take the image.

### 3.1. Three-dimensional microstructure of an artificial sponge

**Figure 3:**
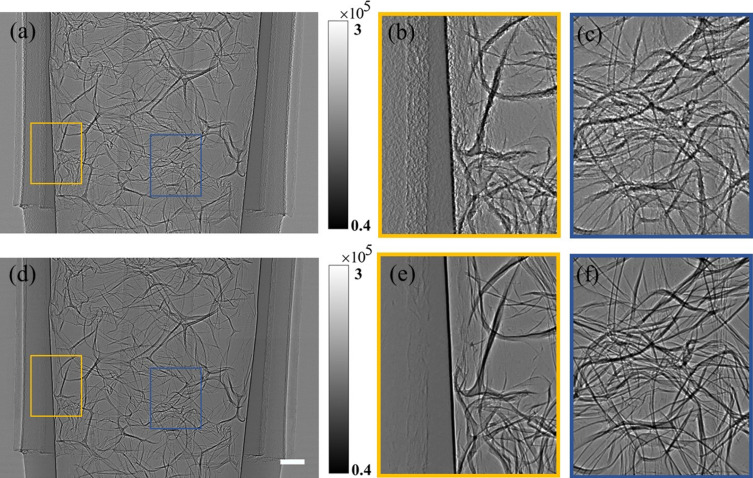
Test results with a phantom of artificial sponge: (*a*) image obtained by subtraction of speckle images with and without sample; (*b*) and (*c*) enlarged images of areas noted in (*a*) with yellow and blue frames, respectively; (*d*) image reconstructed by CGAN; (*e*) and (*f*) are the corresponding enlarged images of areas hghlighted in (*d*) with frames. The scale bars at the right corner of the original image are 500 µm.

An artificial sponge, which is usually used to protect instruments from vibration or shock during transportation, is employed to evaluate the imaging method. The sponge is made of polymers and full of air chambers, in which the chamber walls act as pure phase objects and their interface with the air acts as locations with abrupt changes of density. The artificial-sponge-filled PE tube was selected as a pure phase phantom to demonstrate the capabilities of the proposed method. Fig. 3 ▸ demonstrates how to generate in-line phase contrast images from the original set of speckle images of the sample via deep learning. The test input image of CGAN shown in Fig. 3 ▸(*a*) is an image obtained by image subtraction of speckle images with and without sample, which is in fact the transmission image reconstructed by the traditional speckle-tracking imaging method, taking a subset size of one pixel (Zdora, 2018 ▸). As shown in Figs. 3 ▸(*b*) and 3 ▸(*c*), *i.e.* the enlarged images of areas noted in Fig. 3 ▸(*a*) with yellow and blue frames, respectively, it is obvious that artifacts attributed to the speckle modulation severely deteriorate the image contrast at locations with abrupt changes in density. Fig. 3 ▸(*d*) shows the in-line phase contrast images achieved by deep learning with CGAN, and Figs. 3(*e*) and Fig. 3(*f*) are the enlarged images of areas noted in Fig. 3 ▸(*d*) with frames. As shown in Figs. 3(*e*) and 3(*f*), the fine structures of the sponge are revealed in the in-line phase contrast image retrieved by CGAN and the artifacts attributed to speckle displacement are successfully eliminated. Hereafter, we use the CGAN-generated in-line phase contrast image for further phase retrieval based on the double-exposure method. As for the efficiency in retrieving an in-line phase contrast image by deep learning, the time needed for a single frame with an INVIDIA GeForce RTX 2080Ti GPU is 200 ms.

**Figure 4:**
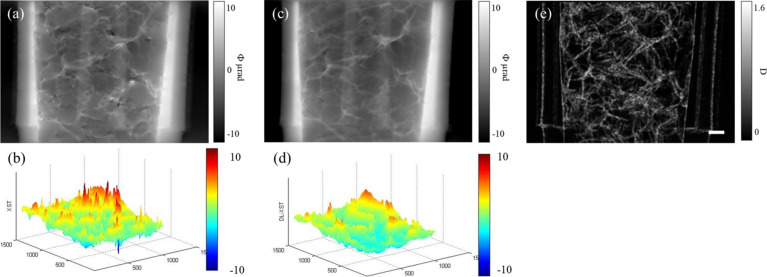
Retrieved phase images of the sponge phantom, reconstructed by the (*a*) standard speckle-tracking method and (*c*) deep-learning based method. (*b*) and (*d*) 3D contours of the phase distribution in (*a*) and (*c*), respectively. (*e*) Dark-field image reconstructed by the deep-learning based method. The scale bars in the right corners are 500 µm.

Then, the in-line phase contrast image achieved by deep learning is used to eliminate the phase distortion according to the double-exposure method. Fig. 4 ▸ shows the results of the retrieved phase images. As a comparison, the phase contrast image retrieved by the traditional speckle-tracking method is also given, as shown in Fig. 4 ▸(*a*). Due to the overlap of air chambers of the sponge in the projection, the test sample is in fact a complexity with plenty of microstructures to be discerned. From Fig. 4 ▸(*a*), it is obvious that phase distortion appears frequently in the retrieved image at locations with an abrupt change in density, *i.e.* the polymer–air interface. Shown in Fig. 4 ▸(*c*) is the phase image retrieved with the deep-learning based method and phase distortions are eliminated effectively compared with Fig. 4 ▸(*a*), which demonstrates the capability of the proposed method for phase contrast imaging of a sample with a complex structure. To reveal the effect of phase restoration explicitly, 3D contours of the phase are given in Figs. 4 ▸(*b*) and 4 ▸(*d*) which correspond to Figs. 4 ▸(*a*) and 4 ▸(*c*), respectively. Fig. 4 ▸(*b*) shows the 3D distribution of the retrieved phases by the traditional speckle-tracking method and the saltation points shown as sharp peaks in deep red or deep blue can be found scattered over the whole phantom of the sponge. These saltation points shown in Fig. 4 ▸(*b*) mean that the phase is not retrieved accurately due to the abrupt change in density at the interface of the polymer and the air inside the sponge. As a comparison, after artifact removal by deep-learning based methods, the results shown in Fig. 4 ▸(*d*) demonstrate that the phase distortion is eliminated effectively. With successful removal of the phase distortion, the dark image of the sponge phantom is also achieved, as shown in Fig. 3 ▸(*e*), in which the outline of the sponge structure is distinguishable based on X-ray scattering at the walls of the air chamber. Due to the overlapping of the multilayers in a thick sample like the sponge, it is hard to demonstrate the effect of phase distortion distinctly with a single project image. Tomography is needed to confirm the ability of the proposed method to retrieve the microstructure of the sponge accurately.

**Figure 5:**
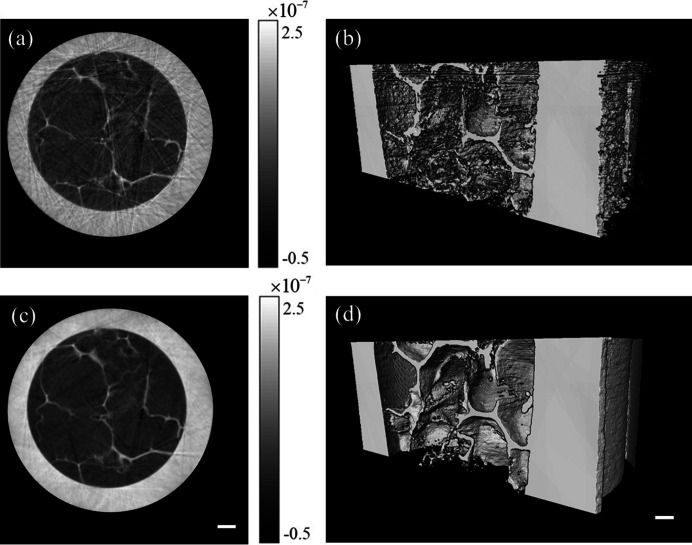
CT images of the artificial sponge reconstructed with the real part of the refractive index: (*a*) slice reconstructed by the traditional speckle-tracking method; (*b*) longitudinal section of the corresponding 3D image; (*c*) and (*d*) results of the deep-learning based method corresponding to the slice and 3D image, respectively. The scale bar represents 500 µm.

To achieve tomographic reconstruction of the artificial sponge, a total of 360 projections were collected over 180° with a step of 0.5°. After phase retrieval, the projections are reconstructed by the FBP algorithm with the Shepp–Logan filter in the *PITRE* software (Chen *et al.*, 2012 ▸). The results of the tomographic reconstruction after phase retrieval are shown in Fig. 5 ▸. Fig. 5 ▸(*a*) shows the slice of the 3D image reconstructed via the traditional speckle-tracking method, and Fig. 5(*b*) is the corresponding longitudinal section. Notably, the radial artifacts in Fig. 5(*a*) severely deteriorate the contrast of the sponge wall. As a result, it is hard to distinguish the air chamber of the sponge separately due to the severe interference of the artifacts as shown in Fig. 5 ▸(*b*). According to the results shown in Fig. 4(*a*), Fig. 4(*c*) and the principle of CT, these artifacts can be attributed to the phase distortion at locations with abrupt changes in density, *i.e.* the interface of the sponge wall and the air. The same sample is also reconstructed with the deep-learning based method. As shown in Fig. 5 ▸(*c*), the radial artifacts are evidently eliminated and the wall of the sponge is revealed in high clarity. Similarly, the longitudinal section of the 3D image is also given in Fig. 5 ▸(*d*). It is evident that the nature of the material interface region of the sponges is successfully revealed and an abrupt, smooth interface between the polymer and air chamber of the artificial sponge is depicted explicitly, compared with the results shown in Fig. 5 ▸(*c*). Accordingly, the tomography results demonstrate that accurate phase retrieval with a single speckle image of the test object is achieved by the proposed deep-learning based method. This suggests that dynamic X-ray phase contrast imaging with high-accuracy phase retrieval is technically feasible via speckle tracking.

### 3.2. Accuracy calibration of phase retrieval

**Figure 6:**
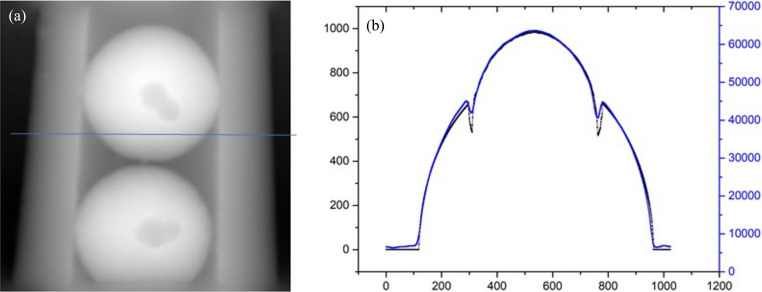
Calibrating the accuracy of the phase retrieval by the deep-learning based method with a phantom: (*a*) retrieved phase image; (*b*) line profile of the retrieved phase image at positions denoted in (*a*) with a blue line, with the theoretical profile supplied for a comparison according to the known size of the phantom.

Furthermore, an artificial phantom is employed to calibrate the accuracy of phase retrieval by a deep-learning based method. Solid spheres of polyoxymethyl­ene (POM) filling a polyethyl­ene (PE) tube comprise the phantom, and the nominal density of the materials for the spheres and tube are 1.41 and 0.945–0.96 g cm^−2^, respectively. Accordingly, the phase profile of the phantom at an energy of 15 keV of the incident X-ray beam can be obtained theoretically based on the size and refractive index of the materials. Fig. 6 ▸(*a*) is the phase image retrieved from a single projection of the phantom. To quantitatively investigate the phase-retrieval accuracy, the profile at positions denoted with a line in Fig. 6 ▸(*a*) is given in Fig. 6 ▸(*b*) together with the corresponding theoretical profile supplied for comparison. From Fig. 6 ▸(*b*), it is obvious that the retrieved profile is highly consistent with the theoretical one. Even for positions with an abrupt change of phase, the trends of the profile retrieved by the proposed method is in accord with the theoretical prediction. Therefore, we can conclude that high-accuracy phase retrieval can be achieved by the proposed method from a single speckle image of the object. This means that the deep-learning based method has the potential for dynamic characterization of the microstructural evolution in low-*Z* materials. Combined with dynamic CT, it is also possible to reveal the 3D evolution of microstructures inside phase objects such as polymers, light alloys and biomaterials.

### 3.3. Dynamic imaging of a foaming process

Polyurethane foaming is selected to evaluate the practicability of the proposed method for dynamic X-ray imaging. The 3D stack of bubbles during foaming results in a complicated microstructure. Obviously, the polyurethane foam is a pure phase object and has significant edge-enhancement effects at the polymer–air interface during in-line phase contrast X-ray imaging. This means that phase distortion by the traditional speckle-tracking method will severely deteriorate the precise revealing of the bubbles. During the experiments, the black and white materials for polyurethane foaming were poured into a PE tube and then stirred evenly to trigger the foaming process. Then the speckle images of the dynamic evolution of bubbles were recorded by an X-ray detector with a frame rate of 20 frames s^−1^ and the exposure time for a single frame is 50 ms.

**Figure 7:**
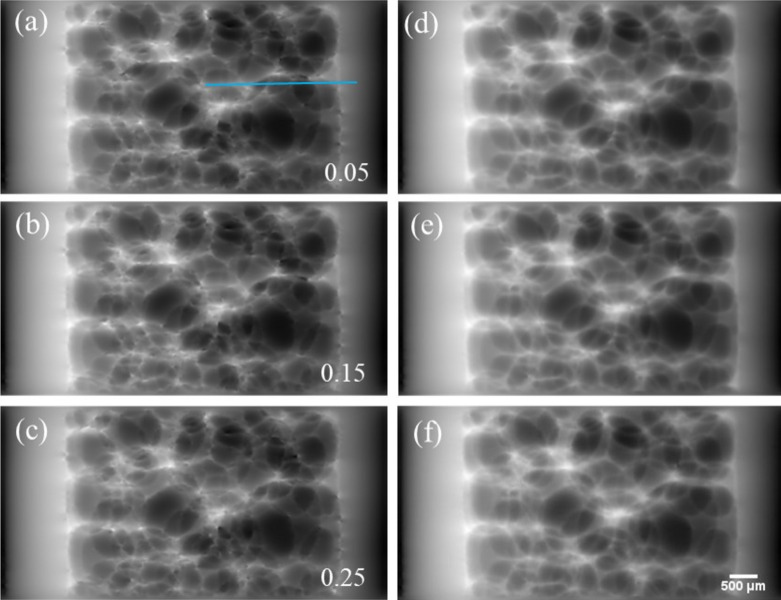
Dynamic imaging of polyurethane foaming: (*a*)–(*c*) projections obtained by the traditional speckle-tracking method at 50, 150, 250 ms, respectively; (*d*)–(*f*) counterpart projections retrieved with the deep-learning based method. Scale bar at the lower right corner is 500 µm.

After data acquisition for the dynamic process of polyurethane foaming, phase retrieval was implemented by means of traditional speckle-tracking and deep-learning based methods, and the results are shown in Fig. 7 ▸. To evaluate the time-resolving ability of the proposed method, three frames recorded at the initial stage of foaming are selected. Figs. 7 ▸(*a*), 7 ▸(*b*) and 7 ▸(*c*) show the phase-retrieval results of the traditional speckle-tracking method, corresponding to time nodes of 50, 150 and 250 ms, respectively. Similar to Fig. 4 ▸(*a*), phase distortion is observed scattered throughout the image field in Figs. 7 ▸(*a*)–7 ▸(*c*). As a result, edges of the bubbles in the foaming stack are obscured, especially for the bubbles with multiple stacks in the direction of the incident X-rays. Accordingly, the proposed method is employed to eliminate the effect of phase distortion and the corresponding results are shown in Figs. 7 ▸(*d*)–7 ▸(*f*), respectively. It is obvious that the phase distortion is eliminated effectively by the deep-learning based method. Without the disturbance of artifacts, the complicated profiles of the bubble stack are depicted distinctly.

**Figure 8:**
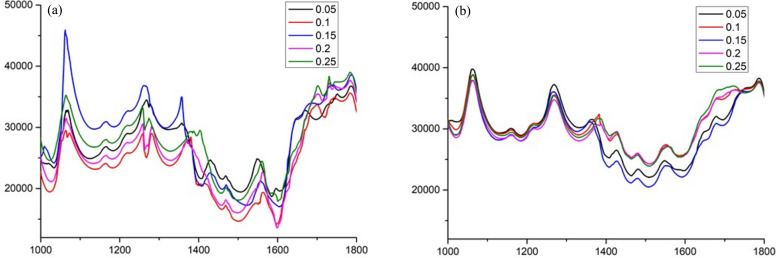
Line profiles of the phase images at time nodes of 50, 100, 150, 200, 250 ms: (*a*) traditional speckle-tracking method, (*b*) deep-learning based method.

During the experiments, the frame rate of data acquisition is 20 frames s^−1^ and the foaming process is relatively slow. According to the direct observation of the images given in Fig. 7 ▸, it is hard to identify significant change among all the well retrieved images collected on the time scale of 250 ms. Therefore, profiles of the retrieved phase at positions marked with a light blue line with a length of 800 pixels from (1000, 592) to (1800, 592) are given in Fig. 8 ▸ to investigate the evolution of the bubbles at time nodes of 50, 100, 150, 200, 250 ms. The phase evolution retrieved by the traditional speckle-tracking method is shown in Fig. 8 ▸(*a*). Due to the interference of phase distortion, obvious phase fluctuation can be observed especially in the range of pixel Nos. 1000–1350. In addition, sharp peaks appeared frequently in the profiles due to phase distortion at positions with density saltation, *i.e.* the polyurethane–air interface. This implies that it is hard to measure the phase evolution quantitatively with the traditional speckle-tracking method. The corresponding results of the deep-learning based method are given in Fig. 8 ▸(*b*), in which profiles of the retrieved phase are smooth overall and the sharp peaks in Fig. 8 ▸(*a*) are eliminated. This is consistent with the nature of a piece of polymer sponge. With the successful elimination of phase distortion, quantitative evaluation of phase evolution by the proposed method is conceivable. According to Fig. 8 ▸(*b*), only a slight variation occurs in the profiles of the retrieved phase from (1000, 592) to (1300, 592) within the time duration of 50–250 ms, which is consistent with the qualitative analysis of the phase images shown in Figs. 7 ▸(*d*)–7 ▸(*f*). As for the profiles in the range from (1745, 592) to (1800, 592), which is close to the inner wall of the container, only minute phase changes were observed on the contrary to the significant fluctuation retrieved by the traditional speckle-tracking method as shown in Fig. 8 ▸(*a*). From (1300, 592) to (1745, 592), an apparent phase change is resolved by the proposed method, which means that the foaming process in this range is more intense than the other ranges investigated. Certainly, all the characteristics of the phase evolution cannot be revealed by the traditional speckle-tracking method. Videos of the whole foaming process are provided in the supporting information, in which the dynamic evolution of the bubbles retrieved by the traditional speckle-tracking method (Viideo S1) and the deep-learning based method (Video S2) can be investigated directly. The experimental results imply that the deep-learning based method is capable of quantitatively characterizing the structure evolution of phase objects.

## 4. Conclusions

We report a method for dynamic X-ray speckle-tracking imaging with high-accuracy phase retrieval based on deep learning. Our approach is in fact a deep-learning based method that uses the CGAN to generate the in-line phase contrast image from a single speckle image of the object, which needed to be collected separately using the double-exposure method (Wang *et al.*, 2017 ▸; Yu *et al.*, 2021 ▸). This means that dynamic X-ray imaging with high-accuracy phase retrieval can be achieved by the deep-learning based method. Calibration with a phantom shows that the profile of the retrieved phase is highly consistent with the theoretical prediction. Dynamics of polyurethane foaming were employed to demonstrate the proposed method and the results show that the evolution of the complicated microstructure of the bubbles was revealed accurately by the deep-learning based speckle-tracking method. In conclusion, the dynamic X-ray speckle-tracking imaging with high-accuracy phase retrieval is achieved by the proposed deep-learning based method.

As for the CGAN training process used in the proposed method, it is usually data hungry and time-consuming. However, it requires only one training effort. Once the CGAN parameter is established, we no longer need to retrain the network. For the test sample, we used the training set of a sample which is of the same type as the test sample but with a different structure in a targeted manner. The results show that only one set of projections of the sample from different angles are needed to train an effective network. This paper focuses on the accuracy of phase retrieval of the proposed method and all the demonstrations are performed on pure phase objects. The effect of absorption on artifact elimination requires further investigation. In general, the network of deep learning can work well with test samples that belong to the types of samples trained previously. Therefore, we believe that adding different types of pure phase samples to the training set can increase the versatility of the network for low-*Z* materials. This means that the proposed method can find extensive applications in metrology and quantitative analysis of dynamics in material science, physics, chemistry and biomedicine.

## Supplementary Material

Visualization 1, dynamic evolution of the bubbles retrieved by traditional speckle-tracking method. DOI: 10.1107/S2052252523010114/ti5027sup1.avi

Visualization 2, dynamic evolution of the bubbles retrieved by deep-learning based method. DOI: 10.1107/S2052252523010114/ti5027sup2.avi

Supporting figures. DOI: 10.1107/S2052252523010114/ti5027sup3.pdf
