# Supplementary material for: Dynamic X-ray speckle-tracking imaging with high-accuracy phase retrieval based on deep learning
Source: IUCrJ. 2024 Jan 1;11(Pt 1):73–81. doi: 10.1107/S2052252523010114 (PMC10833393; doi:10.1107/S2052252523010114)
Supplement: Supplementary file 3 [file m-11-00073-sup3.pdf]

IUCrJ

Volume 11 (2024)

Supporting information for article:

Dynamic X-ray speckle-tracking imaging with high accuracy of phase-retrieval based on deep learning

Fucheng Yu, Kang Du, Xiaolu Ju, Feixiang Wang, Ke Li, Can Chen, Guohao Du, Biao Deng, Honglan Xie and Tiqiao Xiao

S1. Comparison with double exposure method

A comparison between double exposure method and deep-learning based method is supplied. Figure 1S shows the comparison between the double-exposure method and deep-learning based method. Figure 1S(a) illustrates the phase image reconstructed by double exposure method, while Figure 1S(c) displays the line profile corresponding to position denoted with the blue line in Figure 1S(a). Moreover, Figure 1S(b) and Figure 1S(d) refer to the correspondent results of deep-learning based method. As to the line profiles, results of the two methods are quite close to the actual profile, while reconstruction accuracy of the deep-learning based method is little better than that of double exposure method. For the double exposure method, misalignment of the two images recorded independently may lead to reconstruction error. As for the deep-learning based method, the in-line phase contrast image is generated directly from the projection image with speckle and the misalignment of the two images can be avoided

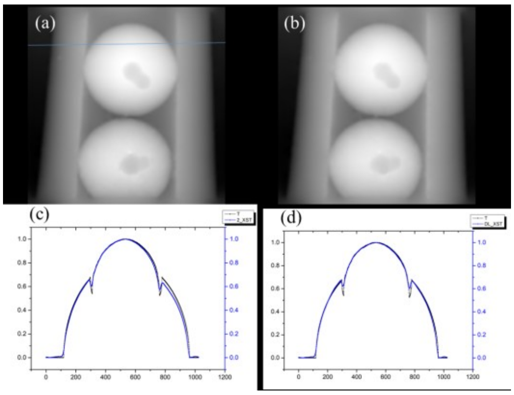

Figure 1S. A comparison between double exposure and deep-learning based methods, where (a) and (b)were phase images reconstructed by double exposure method and deep-learning based method respectively, (c)and (d)were the intensity profile at the position denoted with the blue line in (a).

S2. Data sets for network training

Here are the datasets used for training, with Figs. S2, S3 and S4 showing examples of the training data for artificial sponge, artificial phantom and dynamic polyurethane foaming samples, respectively. The complete dataset can be obtained from the author by email.

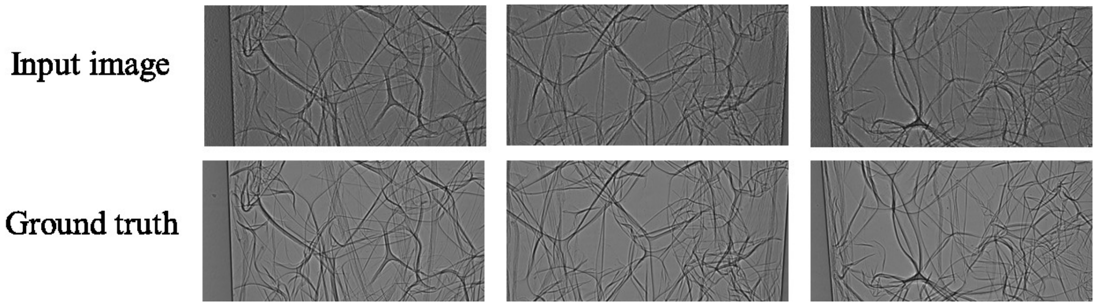

Figure 2S. Examples of training data for artificial sponge .

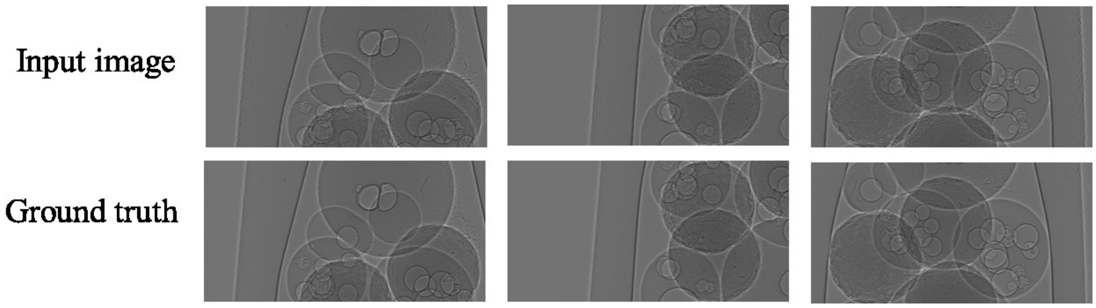

Figure 3S. Examples of training data for artificial phantom.

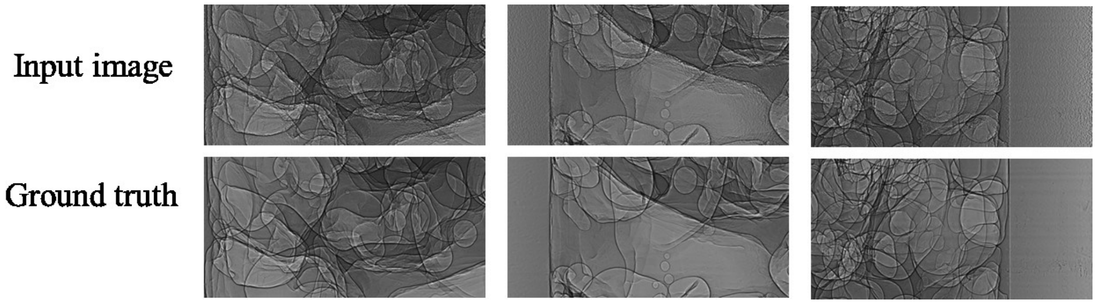

Figure 4S. Examples of training data for dynamic polyurethane foaming.
